# Progress in District Nursing

**Published:** 1907-06-08

**Authors:** 


### Nursing Administration.

### PROGRESS IN DISTRICT NURSING.

There is no more hopeful sign for rural England than the continuous spread of district nursing throughout the country. It is a movement which extends its influence far beyond the sick poor for whose relief it was in the first instance designed. It is not only a source of untold comfort to the sick folk who for want of tendance have hitherto suffered miserably and hopelessly in their own cottages, but also an educative force the full value of which is even now scarcely appreciated except by those who have followed its developments. Reinforced by the midwifery which now forms a part of almost every district nurse's qualifications, it is likely to have a permanent effect on the unborn race, and to introduce wholesome methods of child rearing to combat the old superstitions which have lingered in remote country regions, to the destruction of countless thousands of future citizens. It is bringing the powers of gentle persuasiveness and gentle rebuke to bear against dirt, airlessness, and unconscious cruelty, and among the younger women its effects are already conspicuous in many neighbourhoods in an improved standard of living, and higher ideals of conduct. But the value of district nursing does not end here. It is a bond of union between rich and poor to an extent to which the old district visitor system never attained, much as the country owes to these untrained workers. There are many country districts where the nursing association has brought into being a public spirit and power of co-operating for the common good hitherto unknown. The astonishing result is that even districts where influential support is conspicuously lacking find themselves capable of meeting the heavy annual charges connected with the provision of a trained nurse. The enthusiasm of the poor goes hand in hand with that of the middle classes, and the desire to possess a nurse once awakened in a neighbourhood works irresistibly until the aim is accomplished.

The recent report of the Queen Victoria's Jubilee Institute for Nurses should do much to quicken the cause of district nursing throughout the country. One most important pronouncement has removed a serious impediment from the progress of village nursing—namely, that the statistics "tend decidedly to show that midwifery and non-infectious nursing may be combined with safety by Queen's nurses, or the village nurses of the Institute." So long as this point was in abeyance the village nurse was in a position of considerable difficulty. It was impossible that each village could maintain two nurses, or even that work sufficient for a nurse could be found in small neighbourhoods unless midwifery and the non-infectious cases of general nursing were combined. That the precautions enjoined on the midwives whose duties bring them sometimes into contact with septic cases have proved a sufficient safeguard, is matter for congratulation to those responsible for framing the regulations, and is a tribute to the success of the Midwives Act, which has rendered possible the enforcement of exact rules. The Institute is doing much by taking the village nurse under its ægis through the medium of the county associations. The policy of ignoring the less highly trained nurse seemed at one time likely to be disastrous for the future of nursing, but already much has been done by the introduction into the too short period of training, which is often all that is available, of sound methods, and by regular inspection, to render the village nurse a valuable link in the chain of aid to the sick poor all over the country.

### The Village Nurse.

It would appear now to be only a question of time before the nurse becomes as essential a part of town and country life as the doctor or the parson. Only want of funds stands in the way of her presence in sparsely populated districts, and this, as we have said, is a difficulty which tends to disappear as the want begins to press. The step which is being taken by many Boards of Guardians in making grants for the nursing of the sick poor in their own homes in place of removing them to the workhouse or Poor-law infirmary is likely to become very general as the full extent of the saving effected through the medium of the district nurses is grasped. And would it not be possible for Parish Councils also to vote grants in poor districts for this purpose?

### The Midwife Under the New Act.

The position as regards village nursing will shortly be changed by the disappearance of the *bona fide* midwife, under the regulations of the Midwives Act in 1910. The condition of affairs as regards rural midwifery is grave in the extreme. Few, indeed, are the counties in which any adequate preparation for 1910 is taking place. It is quite hopeless in the present condition of affairs to expect that midwifery in country districts can be undertaken in accordance with the ordinary laws of supply and demand. The magnitude of the task can be best appreciated by contrasting the population of Great Britain, as ascertained at the last census, with the number of nurses available for the service of the poor. In connection with the Jubilee Institute there were on December 31, 1906, a total number of 2,190, including Queen's nurses, probationers, village nurses, and midwives. There are probably as many more district nurses working for associations not affiliated to the Institute. Whereas to complete the nursing service the best authorities agree there should be at the very least one nurse to every 2,000 persons.

Under the present system there are many places without a nurse, which are perfectly able to cope with the necessary expense. We believe these would be much fewer if only the need were brought clearly home to the inhabitants. Could not the Jubilee Institute prepare a Report from the knowledge at their disposal stating the requirements of each county? The organisation is in their hands, and it is to them that the country looks to deal with the matter as a whole.

